# Good-night (Poetry)

**Published:** 1881-08

**Authors:** 


### GOOD NIGHT.

Good night—the little lips touch ours,
  The little arms enfold us;
And oh, that thus through coming years
  They might forever hold us.

Good night! we answer back and smile,
  And kiss the drooping eyes;
But in our trembling hearts the while
  The wistful queries rise—

Who, in the weary years to come,
  When we are hid from sight,
Will clasp these little hands and kiss
  These little lips "Good night?"

A NEW French appliance is an electric brake, which is made to operate on the wheelbrake by means of electricity generated during the motion of the train and applied at pleasure.

